# Neoadjuvant therapy for pMMR/MSS locally advanced rectal cancer in the immunotherapy era: current landscape and future perspectives

**DOI:** 10.3389/fonc.2025.1719642

**Published:** 2025-12-03

**Authors:** Benish Farooq, Xingyu Li, Sa Xiao, Xiaojun Liu

**Affiliations:** 1 School of Life Sciences, Lanzhou University, Lanzhou, China; 2 Gansu University of Chinese Medicine First School of Clinical Medical, Lanzhou, China; 3 State Key Laboratory of Herbage Improvement and Grassland Agro-ecosystems, College of Ecology, Lanzhou University, Lanzhou, China; 4 Division of Radiation Oncology, Gansu Provincial Hospital, Lanzhou, China; 5 The Third Hospital and Clinical Medicine School, Lanzhou University, Lanzhou, China

**Keywords:** rectal cancer, pMMR/MSS, immunotherapy, watch and wait approach, pathological complete regression, clinical complete remission, long-course chemoradiotherapy, short-course chemoradiotherapy

## Abstract

Numerous clinical studies indicate that neoadjuvant chemoradiotherapy (NCRT) with immunotherapy can significantly increase pathological complete response (pCR) and clinical complete response (cCR) rates to over 30-60%, substantially higher than the 15-20% observed with conventional NCRT. This allows more patients to become eligible for a Watch-and-Wait (WW) strategy, successfully preserving organ function. Several phase III randomized controlled trials (RCTs) are currently underway. The combination of NCRT and immunotherapy holds promise for breaking the therapeutic impasse in proficient mismatch repair/microsatellite stability (pMMR/MSS) rectal cancer, markedly enhancing tumor regression and the potential for organ preservation. However, challenges remain for NCRT combined with immunotherapy in this population. First of all, there is an unmet need to identify predictive biomarkers for treatment response in pMMR/MSS rectal cancer. Next, treatment protocols require further optimization, specifically in determining the best radiotherapy fractionation schedule, its sequencing with immunotherapy, and the radiotherapy target volume. Otherwise, the phenomenon of “pseudo-residual disease” complicates traditional radiological assessment of cCR and must be overcome. Furthermore, the long-term survival benefits of combining radiotherapy with immunotherapy need further confirmation. This review provided a comprehensive and in-depth view of immunotherapy-based NCRT (iNCRT) in patients with pMMR/MSS rectal cancer and discuss the new opportunities and challenges this strategy presents for achieving organ preservation.

## 1 Background

The treatment strategy for rectal cancer aims to improve survival and quality of life. The conventional treatment for patients with locally advanced rectal cancer (LARC) is neoadjuvant chemoradiotherapy (NCRT) followed by total mesorectal excision (TME) (1). For patients with mid-low rectal cancer, while ensuring oncological radicality, preserving organ function is a primary concern (1). NCRT significantly reduces local recurrence rates, promotes tumor regression, and can lead to a pathological complete remission (pCR), which is associated with a superior prognosis (2). Some patients achieving clinical complete response (cCR) after NCRT can adopt a WW strategy, preserving the organ while maintaining good anal function and quality of life (3). The total neoadjuvant therapy (TNT) approach can increase the pCR rate to approximately 30% and prolong disease-free survival (DFS) (4). However, less than one-third of patients achieve cCR, indicating a significant gap towards the goal of organ preservation via WW/non-operative management (5).

With advances in immunotherapy, some clinical trials exploring immunotherapy-based NCRT (iNCRT) are increasingly being conducted, demonstrating significant tumor regression effects (6). The synergistic effect between radiotherapy and immunotherapy offers the potential to overcome the relative resistance of proficient mismatch repair (pMMR)/microsatellite stability (MSS) rectal cancer to immunotherapy alone, providing a new option for organ preservation and survival benefit (6).

As oncology enters the immunotherapy era, programmed cell death protein 1/programmed cell death protein ligand 1 (PD-1/PD-L1) inhibitors have become first-line standard care for deficient mismatch repair/microsatellite instability-high (dMMR/MSI-H) metastatic colorectal cancer, achieving very high CR rates (75%-100%) and excellent survival outcomes (7). The NICHE-2 study demonstrated that neoadjuvant immunotherapy with ipilimumab and nivolumab is highly effective and provides a viable treatment pathway for patients with dMMR LARC (8). However, dMMR/MSI-H rectal cancer accounts for less than 20% of cases, most of patients have pMMR/MSS tumors, which are insensitive to immunotherapy alone (9). Consequently, enhancing the efficacy of immunotherapy in pMMR/MSS rectal cancer develops a major research focus.

Preclinical studies show that radiotherapy increases the release of tumor-specific antigens (10). It also promotes the expansion of dendritic cells (DCs) and enhances their antigen presentation capability, thereby activating tumor-specific T cells (10). Additionally, radiotherapy improves the infiltration of tumor-infiltrating lymphocytes (10). Through these mechanisms, it amplifies immune effects within T cells and promotes an *in-situ* vaccine effect. In some cases, this can even lead to abscopal effects (10). Therefore, radiotherapy and immunotherapy have synergistic effects, and their combination holds promise for overcoming the challenges of immunotherapy in pMMR/MSS rectal cancer (11). Nowadays, an increasing number of researchers are exploring NCRT combined with PD-1/PD-L1 inhibitors for the neoadjuvant treatment of pMMR/MSS rectal cancer, with encouraging short-term efficacy results. For patients with mid-early rectal cancer who desire sphincter preservation but for whom it is difficult with traditional surgery, NCRT combined with immunotherapy tended to achieve high CR rate, enabling organ preservation through WW and selective local excision (LE). This article reviews the progress in clinical research on iNCRT for rectal cancer and discusses the prospects of immunotherapy in organ preservation for pMMR/MSS rectal cancer.

## 2 A series of neoadjuvant chemoradiotherapy combined with immunotherapy for LARC

Numerous clinical trials of NCRT combined with PD-1/PD-L1 inhibitors have reported results (**Table 1**), and several Phase III studies are ongoing (**Table 2**). The primary endpoints of published studies are mostly short-term efficacy measures, such as CR rate or tumor regression grade (TRG) (12). These studies vary in designs, including different radiotherapy fractionation (long-course vs. short-course), sequencing of radiotherapy and immunotherapy (induction, concurrent, or consolidation), and cycles of combined immunotherapy and chemotherapy (13). The current iNCRT strategies for LARC can be classified into three main categories for discussion: (1) LCRT combined with immunotherapy monotherapy; (2) LCRT combined with chemotherapy and immunotherapy (chemoimmunotherapy); and (3) SCRT followed by chemotherapy and immunotherapy.

**Table 1:** Clinical studies on immunotherapy based neoadjuvant chemoradiotherapy for pMMR/MSS locally advanced rectal cancer.

Category	Study name	Time (Year)	Region	Study type	Participants size (n)	Patient features	Study design	pCR/cCR rate	Organ preservation rate
LCRT Combined with Immunotherapy Monotherapy	VOLTAGE-A (NCT02948348)	2019	Japan	Phase Ib	37	Stage III: 23%	LCRT → 5x Nivolumab	pCR rate: 30%	NA
	NSABP FR-2 (NCT03102047)	2021	USA	Phase II, Single-Arm	45	Stage III: 89%	LCRT → 4x Durvalumab	pCR Rate: 22.2% cCR Rate: 31.1%	71.4%
	PANDORA (NCT04083365)	2021	Italy	Phase III, Single-Arm	55	T3~4: 95%; N+: 79%	LCRT → 3x Nivolumab	pCR Rate: 34.5%	NA
	AVANA (NCT03854799)	2021	Italy	Phase II, Single-Arm	101	Stage III: 94%	LCRT + 6x Avelumab	pCR Rate: 23%	NA
	R-IMMUNE (NCT03127007)	2023	North America	Phase Ib/II, Single-Arm	37	Stage III: 84%	LCRT + 4x Atezolizumab	pMMR pCR Rate: 24%	NA
	NECTAR (NCT04911517)	2021	China	Phase II, Single-Arm	50	T3~4: 92%; N+: 64%	LCRT + 3x Tislelizumab	pCR Rate: 40%	89.1%
	POLAR-STAR (NCT05245474)	2023	China	Phase II, RCT	186	cT3~4aN0M0 cT1~4aN1~2M0	Arm1: LCRT + 3x Tislelizumab Arm2: LCRT → 3x Tislelizumab Arm3: LCRT	pCR Rate Arm1: 27.1% Arm2: 32.7% Arm3: 14.0%	Arm1: 88% Arm2: 87% Arm3: 70%
LCRT Combined with Chemotherapy and Immunotherapy	PKUCH 04 (NCT04340401)	2022	China	Phase II, Single-Arm	25	N2: 76%; MRF+: 56%	3x (CAPOX + Camrelizumab) → LCRT + 2x CAPOX	pCR Rate: 28% WW (cCR+ncCR): 16%	72%
	DUREC (NCT04293419)	2023	Spain	Phase II, Single-Arm	62	T3~4/N+	6x FOLFOX + 6x Durvalumab → LCRT	pCR Rate: 39%	NA
	CHOICE-I (NCT03856411)	2025	China	Phase II, Single-Arm	23	ultra-low rectal cancer with stages T1-3a N0-1	LCRT + 2x Sintilimab → 6x (CAPOX + Sintilimab)	cCR/ ncCR rate: 65.2%	95.5%
	STARS-RC03 (NCT04906044)	2023	China	Phase II, Single-Arm	30	T3~4/N+	TNT + Sintilimab	pCR Rate: 55%	NA
	NRG-GI002 (NCT02921256)	2021	USA	Phase II, RCT	95	High-risk T3~4/N+	Arm1: FOLFOX 6 → (LCRT + 6x Pembrolizumab) Arm2: FOLFOX 6 → LCRT	pCR Rate Arm1: 31.9% Arm2: 29.4%	Arm1: 59.4% Arm2: 71.0%
	SYSUCC Study (NCT04304209)	2019	China	Phase II, RCT	134	T3~4/N+	Arm1: (Sintilimab + CAPOX) → 50Gy RT Arm2: CAPOX → 50Gy RT	cCR Rate Arm1: 44.8% Arm2: 26.9%	Non-Sphincter Surgery Rate Arm1: 6% Arm2: 3%
SCRT Followed by Chemotherapy and Immunotherapy	Averectal (NCT03503630)	2021	Europe	Phase II, Single-Arm	40	Stage III: 91%	SCRT → 6x (FOLFOX + Avelumab)	pCR Rate: 37.5%	NA
	Union Hosp. II (NCT04231552)	2021	China	Phase II, Single-Arm	30	High-risk T3/N2	SCRT → 2x (CAPOX + Camrelizumab)	pMMR pCR Rate: 46.2%	88.9%
	PRECAM (NCT05216653)	2023	China	Phase II, Single-Arm	32	T3/N+	SCRT → CAPOX 2 + 6x Envafolimab (once weekly)	pCR Rate: 62.5%	NA
	TORCH (NCT04518280)	2023	China	Phase II, RCT	130	T3/N+	Arm A (Consolidation): SCRT → 6x (CAPOX + Toripalimab) Arm B (Induction): 2x (CAPOX + Toripalimab) → SCRT → 4x (CAPOX + Toripalimab)	cCR Rate Arm A: 56.5% Arm B: 54.2%	Arm A: 82.3% Arm B: 86.4%
	SPRING-01 (ChiCTR2100052288)	2021	China	Phase II, RCT	98	High-risk T3/N+	Arm1: SCRT → 6x (CAPOX + Sintilimab) Arm2: SCRT → CAPOX 6 (TNT)	cCR/pCR Rate Arm1: 59.2% Arm2: 32.7%	NA
	UNION (NCT04928807)	2023	China	PhaseIII, RCT	231	T3/N+	Arm1 (CAM+CAPOX): SCRT → 2x (CAPOX + Camrelizumab) Arm2 (CAPOX): LCRT → 2x CAPOX	pCR Rate Arm1: 39.8% Arm2: 15.3%	Arm1: 94.2% Arm2: 89.9%
	STELLAR II (NCT05484024)	2023	China	Phase II/III, RCT	588	T3/N+	Arm1 (iTNT): SCRT → (4x CAPOX / 6x FOLFOX) + 4x Sintilimab Arm2 (TNT): SCRT → 4x CAPOX / 6x FOLFOX	cCR Rate Arm1: 45.5% Arm2: 25.0%	NA
LCRT vs. SCRT with Chemotherapy and Immunotherapy	PRIME-RT (NCT04621370)	2020	UK	Phase II, RCT	48	cT3b-4b/N+, EMVI+ or Low Position	Arm1 (SCRT): (SCRT → FOLFOX) + Durvalumab Arm2 (LCRT): (LCRT → FOLFOX 4) + Durvalumab	cCR Rate Arm1: 57% Arm2: 48%	NA

EMVI, Extramural Vascular Invasion; cCR, clinical complete response; LARC, locally advanced rectal cancer; LCRT, long-course chemoradiotherapy; ncCR, near clinical complete remission; NCRT, neoadjuvant chemoradiotherapy; pCR, proficient mismatch repair; pMMR, randomized controlled trials; RCTs, short-course radiotherapy; TNT, total neoadjuvant therapy.

**Table 2:** Ongoing phase III clinical studies on immunotherapy based neoadjuvant chemoradiotherapy for pMMR/MSS locally advanced rectal cancer.

Category	Study name	Country	Study type	Participants size (n)	Patient characteristics	Study design	Primary endpoint
SCRT Followed by Chemotherapy and Immunotherapy	mRCAT-III (NCT06507371)	China	PhaseIII	170	T3-4, N0-2	Arm1: SCRT (Tumor+Nodes) → 4x (CAPOX + Sintilimab) Arm2: SCRT (Tumor Only) → 4x (CAPOX + Sintilimab) Arm3: SCRT → 4x CAPOX	pCR, cCR
LCRT Combined with Chemotherapy and Immunotherapy	CHOICEII (NCT05215379)	China	PhaseII-III	180	≤ 5cm from anal verge; T2, N1-2, M0	Arm1: LCRT → 2x CAPOX Arm2: LCRT + 2x Sintilimab → 2x (CAPOX + Sintilimab)	cCR
	Beijing Friendship Hospital (NCT06312982)	China	PhaseIII	375	T3-4, N0-2, M0	Arm1: LCRT → 2x CAPOX Arm2: LCRT + 3x Sintilimab → 2x CAPOX	cCR
	PKUCH-R07 (NCT06229041)	China	PhaseIII	472	T4b, N2, MRF+, EMVI+, LLN+	Arm1: TNT Arm2: TNT + Immunotherapy	pCR

cCR, clinical complete response; LARC, locally advanced rectal cancer; LCRT, long-course chemoradiotherapy; pCR, pathological complete response; RCTs, randomized controlled trials; SCRT, short-course radiotherapy; MRF, Mesorectal Fascia; TNT, total neoadjuvant therapy.

### 2.1 LCRT combined with immunotherapy monotherapy for LARC

LCRT combined with immunotherapy monotherapy yields CR rates around 30%, as seen in studies with sequential radiotherapy and immunotherapy like VOLTAGE-A (14), NSABP FR-2 (15), PANDORA (16), and studies with concurrent LCRT and immunotherapy like ANAVA (17), R-IMMUNE (18), CHOICE-I (19), and NECTAR (20). These rates are higher than standard NCRT (15%-20%) (21).

In POLAR-STAR study, LARC patients were randomized into three groups: LCRT concurrent with PD-1 antibody (3 cycles), LCRT followed by PD-1 antibody (3 cycles), and LCRT alone (control group). Both experimental groups showed higher pCR rates compared to the control group (33.9% and 34.5% vs 15.5%, P = 0.021 and 0.019) (22). The results strongly support the biological rationale that PD-1 blockade can augment the anti-tumor immune response primed by radiotherapy. It moves the field from “proof-of-concept” to “proof-of-efficacy” in a randomized setting. However, the interpretation of the study’s findings must consider its inherent limitations, including its Phase II design and limited sample size.

### 2.2 LCRT combined with chemotherapy and immunotherapy For LARC

A Phase II RCT conducted of LCRT combined with chemotherapy and a PD-1 inhibitor (NCT04304209) (23). The experimental arm received one cycle of CAPOX plus PD-1 antibody induction, followed by LCRC with concurrent two cycles of CAPOX plus PD-1 antibody and one additional cycle of the combination after radiotherapy. The control arm received one cycle of CAPOX induction, followed by long-course radiotherapy with concurrent two cycles of CAPOX, and one additional cycle of CAPOX post-radiotherapy. Patients achieving cCR received four more cycles of CAPOX before entering WW; those not achieving cCR (non-cCR) were recommended for TME, followed by four cycles of CAPOX. The primary endpoint was CR rate. Results showed a significantly higher CR rate in the experimental group (44.8% vs. 26.9%; P = 0.031, RR = 1.67). The non-sphincter-preserving surgery rates were 6% and 3% in the experimental and control groups, respectively (23). This study tests a powerful combination of immunotherapy, chemotherapy (CAPOX), and radiotherapy in a coordinated sequence. This also reflects a modern trend towards intensive, systemic therapy-first approaches. The primary limitations of this study include the constrained generalizability of its Phase II design and the unanswered question of long-term survival benefit, as the data remain immature.

CHOICE-I study evaluated the efficacy and safety of iTNT. It was a single-arm Phase II trial including 23 patients with T1-3a N0–1 ultra-low rectal cancer, of which 69.6% were at N0 stage. This study employed LCRT combined with two cycles of sintilimab, followed by six cycles of sintilimab plus CAPOX (capecitabine + oxaliplatin). Among them, 10 and 5 patients achieved cCR and ncCR (near clinical complete remission), respectively. The rectum and anus preservation rates were 63.4% (14/22) and 95.5% (21/22), respectively (19). This study represents an innovative attempt to integrate immunotherapy into the standard-of-care for LARC. Its findings have generated considerable interest and set the stage for the next generation of clinical trials. The primary limitations of the CHOICE-I study are due to its single-arm, non-randomized design and its small sample size.

The NRG-GI002 study was a phase II randomized clinical trial platform investigating the addition of pembrolizumab to a TNT backbone for high-risk LARC patients (24). The experimental arm received four months of neoadjuvant FOLFOX followed by long-course chemoradiotherapy (50.4 Gy with capecitabine) concurrently with pembrolizumab (200 mg every 3 weeks for up to 6 doses). The primary endpoint was the Neoadjuvant Rectal (NAR) score. Results showed that the addition of pembrolizumab did not significantly improve the mean NAR score compared to the control TNT arm (11.53 vs. 14.08; P = 0.26). Furthermore, no significant differences were observed in key secondary short-term efficacy endpoints, including the pCR rate (31.9% vs. 29.4%; P = 0.75) and cCR rate (13.9% vs. 13.6%; P = 0.95). The study concluded that while the regimen was safe, the lack of improvement in the NAR score did not support further investigation of this specific TNT-plus-pembrolizumab strategy.

Currently, several Phase III RCTs targeting rectal cancer patients with different risk stratifications are ongoing. These include the PKUCH-R07 study (NCT06229041) for high-risk patients comparing TNT with or without immunotherapy; a study (NCT06312982) focusing on low-risk patients comparing LCRT followed by chemotherapy vs. concurrent immunochemotherapy; and the CHOICE II study (NCT05215379) for ultra-low, low-risk patients evaluating LCRT followed by chemotherapy vs. concurrent immunochemotherapy. These randomized controlled studies aim to assess whether adding immunotherapy to traditional NCRT further improves treatment efficacy.

### 2.3 SCRT followed by chemotherapy and immunotherapy for LARC

SCRT is another widely used neoadjuvant regimen beyond LCRT for rectal cancer. SCRT followed by chemotherapy can achieve pCR rates similar to LCRT. Studies suggest that combining hypofractionated SCRT with immunotherapy may offer advantages over conventional fractionated radiotherapy. Several clinical studies of SCRT combined with chemotherapy and immunotherapy have reported excellent short-term efficacy.

The TORCH study adopted an iTNT model combining SCRT with chemoinmunotherapy and allowed selective WW. This randomized study enrolled 130 patients into consolidation (Group A) and induction (Group B) groups. Group A received SCRT followed by six cycles of CAPOX plus toripalimab (a PD-1 antibody); Group B received two cycles of CAPOX plus toripalimab induction, then SCRT, followed by four cycles of CAPOX plus toripalimab, then TME, with WW option for cCR patients. The overall CR rate was 55.4% (67/121), with 56.5% (35/62) in Group A and 54.2% (32/59) in Group B. The post-neoadjuvant cCR rates were 43.5% (27/62) and 35.6% (21/59) for Groups A and B, respectively, and anal preservation rates were 82.3% (51/62) and 86.4% (51/59), respectively (25). The most significant contribution of this study lies in its high-profile demonstration that a TNT strategy centered on immunotherapy can yield exceptional cCR rates, thereby powerfully expanding the potential for non-operative management.

The Phase II UNION study enrolled high-risk LARC patients using a regimen of SCRT followed by two cycles of CAPOX chemotherapy combined with camrelizumab (a PD-1 antibody). Results showed a pCR rate of 48% (13/27) in the 27 patients who underwent surgery, with a pCR rate of 46% (12/26) in pMMR/MSS patients, and an anal preservation rate of 89% (24/27). Achieving nearly 50% pCR within a 2-month treatment cycle confirms significant synergy between hypofractionated radiotherapy and immunotherapy (26). The UNION study holds significant clinical importance by establishing a novel and highly effective total neoadjuvant therapy paradigm for locally advanced rectal cancer, demonstrating that short-course radiotherapy followed by consolidation chemotherapy and immunotherapy can yield exceptional pathological complete response rates, thereby significantly expanding the potential for organ preservation.

The SPRING-01 study used a similar iTNT regimen of SCRT followed by chemotherapy and immunotherapy, significantly increasing the pCR rate in a high-risk population (59.2% vs. 32.7%; P = 0.015), with a CR rate of 61.2% in the experimental group, significantly better than traditional SCRT followed by chemotherapy (TNT) (27). The design of this study also provides a critical head-to-head evaluation of a modern chemotherapy-based TNT strategy against an immunotherapy-based TNT strategy, both built upon the same SCRT foundation.

SCRT combined with chemotherapy and immunotherapy has demonstrated high CR rates across multiple studies, but its efficacy needs further validation in Phase III RCTs. Based on preliminary results, the Phase III randomized controlled multicenter UNION study was initiated, enrolling 231 patients randomized to CAPOX or CAM (camrelizumab) + CAPOX groups. The CAPOX group received LCRT (50.4Gy with capecitabine) followed by two cycles of CAPOX, then TME and six cycles of CAPOX. The CAM+CAPOX group received SCRT (25Gy/5Fx) followed by two cycles of CAPOX plus camrelizumab, then TME and 6–9 cycles of CAPOX plus camrelizumab. The results showed a significantly higher pCR rate in the CAM+CAPOX group (39.8% vs. 15.3%; P < 0.001), with anal preservation rates of 94.2% and 89.9%, respectively (26).

Furthermore, based on the prior STELLAR study, the Phase II/III RCT STELLAR II study was initiated to compare SCRT combined with chemotherapy and immunotherapy (iTNT model) versus LCRT followed by chemotherapy (TNT model) (NCT05484024). The primary endpoints are CR rate (Phase II) and 3-year DFS rate (Phase III). The team reported preliminary efficacy data for the first 218 patients at the 2025 ESTRO meeting, showing a significantly higher CR rate in the iTNT group (45.5% vs. 25.0%, P = 0.002) (28, 29).

Current data indicate that combining chemotherapy and immunotherapy with either LCRT or SCRT shows significant short-term efficacy, with immunotherapy holding important clinical value for organ function preservation in pMMR/MSS rectal cancer. However, the interpretation of these robust findings is inherently constrained by the study’s single-arm, non-randomized Phase II design and its limited sample size, which preclude definitive conclusions about its superiority over standard regimens and leave the critical question of long-term survival benefit and durability of organ preservation unanswered. The long-term survival benefits require further follow-up evaluation. Moreover, no consensus exists on the optimal combination regimen of radiotherapy, chemotherapy, and immunotherapy, warranting further investigation through large-scale RCTs.

## 3 Exploration of optimal radiotherapy-immunotherapy combination strategies

The comparative value of LCRT and SCRT in rectal cancer neoadjuvant therapy remains a key clinical research topic<no>.</no> In the conventional NCRT era, LCRT demonstrated higher organ preservation rates and lower pelvic recurrence rates compared to SCRT (30); however, their relative merits in the immunotherapy era require further validation. Compared to conventional fractionation, hypofractionated radiotherapy (like SCRT) may achieve superior anti-tumor effect through its multi-faceted immunomodulatory effects. These include promoting tumor antigen release and immunogenicity, as well as stimulating cytokine secretion. Furthermore, it better preserves peripheral blood lymphocytes and inhibits the recruitment of myeloid-derived suppressor cells into the tumor microenvironment. Collectively, these actions enhance the anti-tumor immune response (31). A small clinical study in locally advanced non-small cell lung cancer (n = 45) randomized patients to three radiotherapy-immunotherapy regimens: conventional fractionation (2Gy×20), hypofractionation (5Gy×5), and ultra-hypofractionation (8Gy×3), showing significantly better complete response rates in the hypofractionated and ultra-hypofractionated groups, supporting the potential advantage of hypofractionated radiotherapy combined with immunotherapy (32). A meta-analysis indicated a significantly higher pCR rate for SCRT combined with immunotherapy compared to LCRT combined with immunotherapy (51% vs. 30%) (33). The PRIME-RT study (34), the first RCT comparing short-course and long-course radiotherapy combined with immunotherapy (both using durvalumab anti-PD-L1 antibody in an iTNT model), reported interim results at ESTRO 2025 (NCT04621370). Among 46 enrolled patients, the CR rate of evaluable patients at six months was 67% in the short-course iTNT arm. This a significant improvement compared to previous treatments (34). However, due to the small sample size, the superiority between LCRT and SCRT requires validation in larger studies. The sequencing of radiotherapy and immunotherapy is a critical factor affecting treatment efficacy. In the conventional NCRT era, the consolidation model (radiotherapy followed by chemotherapy) yielded higher organ preservation rates than the induction chemotherapy model (35). However, in the immunotherapy era, the optimal strategy among concurrent, consolidation, and induction remains debated. The POLAR-STAR study showed no significant difference in pCR or organ preservation rates between concurrent and sequential LCRT with immunotherapy monotherapy (36). The TORCH study compared consolidation and induction iTNT schedules; although the difference in overall CR rate was not statistically significant, the absolute post-neoadjuvant cCR rate was higher in the consolidation group (43.5% vs. 35.6%), suggesting a consolidation (radiotherapy-first) approach might be more favorable for organ preservation (25). Notably, despite exploring various schedules, large prospective controlled studies, systematic reviews, and related basic-translational research are still lacking.

According to preliminary studies, elective nodal irradiation (ENI) may systemically decrease the immune response by inhibiting dendritic cell antigen presentation and impairing tumor-specific T cells-steps that are critical for activation, proliferation, circulation, and tumor infiltration—thereby diminishing tumor regression from radiation and immunotherapy (37). Thus, in the immunotherapy era, reducing ENI has potential theoretical advantages. However, for rectal cancer patients, whether reducing pelvic elective nodal target volumes can improve tumor regression rates and survival benefits, or reduce intestinal toxicity, needs further validation through clinical studies (38). The ongoing mRCAT-III study is the first Phase III multicenter RCT exploring reduced ENI in rectal cancer, comparing tumor regression between SCRT (irradiating rectal tumor + nodes) and SCRT to tumor only followed by four cycles of chemotherapy combined with immunotherapy (39). New concepts in radiotherapy strategies are emerging in the immunotherapy era, with radiotherapy combined with immunotherapy gradually showing new transformative directions.

## 4 Challenges and perspectives for standardization of organ preservation assessment after NCRT and immunotherapy

In the evolving landscape of rectal cancer treatment, the demand for organ preservation is becoming increasingly prominent. Studies show that patients achieving cCR after NCRT managed with WW have survival outcomes comparable to those with pCR, alongside better anorectal function and quality of life (40). The goal of neoadjuvant therapy for LARC has shifted from merely controlling local recurrence to enhancing tumor regression, achieving organ preservation, and improving long-term prognosis (41). However, significant heterogeneity exists in the assessment criteria for anal preservation rates across studies, as they employ varied definitions such as anal preservation at surgery, during 1–3 years of follow-up, or non-stoma rates (42). Furthermore, due to variations in neoadjuvant treatment cycles and subsequent decision-making, anal preservation rates vary widely (70%-99%). In most current studies, researchers still regard surgery as the primary treatment option, with relatively limited research implementing WW strategies after TNT or immune-based iTNT (42).

Notably, clinical studies aiming for organ preservation must account for local recurrences within 1–2 years in Watch and Wait patients requiring salvage surgery, necessitating long-term follow-up for accurate organ preservation assessment. Simultaneously, organ preservation involves not just anatomical integrity but also functional maintenance. Existing clinical studies often lack standardized assessment of anorectal function (43). Using professional scales to quantify subjective feelings into objective scores, coupled with standardized follow-up procedures and strict quality control, is recommended to ensure assessment reliability (43).

## 5 Optimization of response evaluation methods after NCRT and immunotherapy

Response assessment after neoadjuvant therapy is crucial for surgical decision-making and Watch and Wait strategy selection in mid-low rectal cancer. However, immunotherapy significantly increases the complexity of evaluating tumor regression (44). Currently, no internationally unified standard exists for neoadjuvant response assessment, primarily relying on digital rectal exam (DRE), MRI, serum tumor markers, endoscopy, and biopsy, supplemented by endorectal ultrasound and PET/CT (42). Importantly, minimal residual disease (MRD) can persist in patients assessed as cCR by traditional methods, while some assessed as non-cCR show pCR on postoperative pathology, known as the “pseudo-residual” phenomenon (45). Studies indicate a 10% pseudo-residual rate in the OPRA study (35) (traditional TNT), rising to 34.2% (25/73) in the TORCH study (25) (immune-based iTNT).

Consistent with other immunotherapy studies, the pseudo-residual phenomenon is more pronounced in the immunotherapy era (46), with a considerable discrepancy rate between endoscopic and MRI assessment (47). Pseudo-residual disease is characterized by two key processes: first, extensive collagen deposition and fibrotic remodeling that creates a mass-like scar (48), and second, a robust and persistent immunoinflammatory response featuring dense lymphocytic infiltration (49) and the potential formation of tertiary lymphoid structures (50). These processes manifest on MRI as T2-weighted signal abnormality, restricted diffusion, and contrast enhancement, while on endoscopy, they present as ulceration, wall rigidity, and edema, all of which are indistinguishable from true residual carcinoma (51). This biological mimicry directly confounds the clinical assessment of a cCR, creating a profound dilemma in “WW” candidate selection (47). Consequently, the risk of either incorrectly offering organ preservation to a patient with residual disease or, conversely, denying it to a patient with a sterilized tumor bed remains unacceptably high with current diagnostic modalities. Future efforts must therefore prioritize the development and validation of advanced, discriminatory biomarkers. These include quantitative imaging radiomics to decode tissue heterogeneity, novel MRI sequences sensitive to specific microenvironmental features, and the integration of liquid biopsies such as circulating tumor DNA analysis to provide a molecular correlate of tumor sterilization, thereby enabling a more precise and personalized selection paradigm (52–54).

## 6 Identification of predictive biomarkers for NCRT with immunotherapy

NCRT combined with immunotherapy has demonstrated significant tumor regression efficacy, but response evaluation and prediction remain challenging. Establishing precise predictive method is clinically crucial for optimizing decision-making (42). Current research efforts focus on analyzing immune microenvironment features, genomic characteristics, and circulating tumor DNA (ctDNA) dynamics to identify potential biomarkers for predicting tumor regression and long-term survival (55).

Regarding the immune microenvironment, the Averectal study (56) found baseline tumor immune score correlated with tumor regression (pCR/TRG). The VOLTAGE (14) study, analyzing tumor tissue at baseline, post-NCRT, and post-3 cycles of immunotherapy, found higher pCR rates in patients with baseline tumor PD-L1 tumor positive score (TPS) ≥1% and dynamically increasing CD8/Treg ratio. The Voltage-A study (57) reported a pCR rate of 75% for TPS 1% and 17% for TPS <1%. The Phase II Union study (58) showed higher pCR rates in patients with baseline tumor PD-L1 combined positive score (CPS) ≥1. A phase II clinical trial also confirmed higher CR rates and greater immune cell infiltration in patients with baseline PD-L1 CPS ≥ 5 compared to <5 (50.0% vs. 27.3%, *P* = 0.311) (59).

In genomic mutation analysis, data from the China SYSUCC study showed that *PDGFA* and *IL2RG* mutations were significantly associated with poorer tumor regression in both traditional NCRT and immunotherapy groups (P < 0.05) (23). Further analysis found *PDGFA* mutation positively correlated with endothelial cell abundance in the TME (r= 0.68; P = 0.003), while *IL2RG* mutation positively correlated with CD4+ regulatory T cell (Treg) abundance (r = 0.72; P = 0.002) (23). Genomic analysis in the PANDORA study (16) indicated that patients with *ARID1A* (17%) or *SMAD4* (21%) mutations did not achieve pCR (non-pCR); *SMAD4* mutation correlated significantly with low PD-L1 expression (P = 0.017). Although 31% of patients had high tumor mutation burden (TMB), no correlation with treatment response was found (P > 0.05) (16). High TMB status correlated significantly with *ARID1A*, *FBXW7*, *MYC*, and *RICTOR* mutations (P < 0.05) (16).

In liquid biopsy, ctDNA shows unique clinical value for detecting MRD post-treatment. Multiple studies indicate the utility of dynamic ctDNA monitoring for response assessment and prognosis prediction in rectal cancer NCRT. The UNION study (60) analyzed 244 plasma samples from 79 patients at baseline (C1), post-radiotherapy (C2), post-chemoimmunotherapy (C3), and post-surgery (C4). ctDNA levels decreased significantly during neoadjuvant therapy. In the short-course immunotherapy group, post-radiotherapy ctDNA clearance (P = 0.049) and MRD clearance (P = 0.015) were significantly associated with pCR. An MRD-based risk score prediction model had best performance at C2 (AUC = 0.85), and patients undetectable for ctDNA-MRD during neoadjuvant therapy had better prognosis. Notably, a prediction model combining MRD clearance and CEA (AUC = 0.983; 95% CI 0.937-1.000) outperformed schedules based solely on MRD clearance (AUC = 0.917) or CEA (AUC = 0.733) for predicting pCR/non-pCR (60). Furthermore, compared to LCRT, SCRT combined with immunotherapy significantly increased tumor tissue MSI (P = 0.042), which might be one mechanism for its enhanced efficacy (60).

## 7 Long-term survival benefits require follow-up after NCRT with immunotherapy

Radiotherapy combined with immunotherapy, by enhancing immune response, theoretically helps induce long-term immune memory, creating a “tail effect” with potential advantages for sustained tumor response and prolonged patient survival (61). Recent studies [including PRODIGE 23 (62), RAPIDO (63), STELLAR (28)] showed that traditional TNT significantly improves tumor regression, reduces distant metastasis risk, and extends survival. However, the long-term survival benefits of NCRT combined with immunotherapy for survival benefit needs further confirmation.

In the realm of LCRT combined with immunotherapy monotherapy, the VOLTAGE-A study (14), the first exploring radiotherapy followed by immunotherapy for LARC, reported long-term follow-up: 3-year recurrence-free survival (RFS) was 79.5% and 3-year OS was 97.4% in pMMR/MSS patients. Subgroup analysis showed better 3-year RFS in patients with baseline PD-L1 positivity (TPS ≥ 1% or CPS ≥ 1%), CD8/eTreg ratio ≥ 2.5, and high expression of Ki67, CTLA-4, PD-1 in CD8+ T cells. Despite promising survival outcomes, due to relatively low treatment intensity and limited sample size, long-term survival benefits require validation in larger studies.

For LCRT combined with chemotherapy and immunotherapy, the focus of clinical trials has often been on intensifying systemic therapy to improve long-term outcomes. The NRG-GI002 study (64) exemplifies this approach, employing a regimen of induction chemotherapy followed by LCRT concurrent with pembrolizumab. While short-term efficacy metrics like pCR were not the primary focus of its long-term analysis, the survival data are highly informative. Long-term follow-up presented at ASCO GI 2023 showed comparable 3-year DFS between the pembrolizumab and control groups (64% vs. 63%), but a superior 3-year OS rate for the pembrolizumab group (95% vs. 87%; P = 0.04), suggesting a potential survival benefit from adding immunotherapy to this intensive TNT backbone. Notably, significant heterogeneity exists in treatment schedules across current studies: NRG-GI002 (64) used induction chemotherapy followed by LCRT concurrent with immunotherapy, while studies like CHOICE-I (19), and PKUCH 04 (65) used concurrent immunotherapy and chemotherapy schemes.

For SCRT followed by chemotherapy and immunotherapy, long-term follow-up data from the Phase II study (58) showed a local recurrence rate of 3.7% (1/27) and distant metastasis rate of 18.5% (5/27) after a median follow-up of 40.8 months. The 3-year DFS and 3-year OS rates were 80.2% and 93.3%, respectively. Subgroup analysis indicated better 3-year DFS and OS in patients achieving pCR, ypN0, MRF negative, EMVI negative, and CPS ≥ 1. However, due to the relatively lower biological effective dose of SCRT, potential local regrowth and pelvic recurrence risks require further assessment through long-term follow-up.

The iTNT model, adding immunotherapy to either long-course or short-course TNT backbone, is primarily applied to high-risk groups. By delivering intensive systemic therapy early, it may further reduce metastasis risk while enhancing tumor regression. Ongoing studies like TORCH (25), STELLAR II (29), and PKUCH-R07 ((NCT06229041) are exploring this strategy, and whether it translates into superior long-term survival benefits is eagerly awaited.

## 8 Summary

For pMMR/MSS LARC patients, NCRT combined with immunotherapy induces significant tumor regression, offering a promising new strategy for organ preservation. However, the organ preservation rates and survival benefits observed in these studies require further validation through long-term follow-up. Future research should focus on conducting large-scale randomized controlled clinical and translational studies, with key priorities including the optimization of radiotherapy-immunotherapy combinations, and the development of precise response evaluation schedules, as well as the identification of effective predictive biomarkers. Furthermore, the consistent implementation of internationally recognized, patient-reported outcome tools, such as the LARS score, will be indispensable for objectively quantifying functional outcomes and comparing results across future clinical trials (66). Through systematic research, the current excellent short-term efficacy may be translated into substantial improvements in organ preservation rates and long-term survival for LARC patients. In the future, integrating high-sensitivity techniques like functional MRI, radiomics, deep learning, and ctDNA-MRD detection with traditional methods is advised to build more accurate response assessment schedules for optimizing surgical and Watch and Wait decisions.
